# Temporal trend in the incidence of tuberculosis-HIV coinfection in Brazil, by macro-region, Federative Unit, sex and age group, 2010-2021

**DOI:** 10.1590/S2237-96222024v33e2023522.en

**Published:** 2024-02-19

**Authors:** Lucas Vinícius de Lima, Gabriel Pavinati, Rosana Rosseto de Oliveira, Rodrigo de Macedo Couto, Kleydson Bonfim Andrade Alves, Gabriela Tavares Magnabosco

**Affiliations:** 1 Universidade Estadual de Maringá, Programa de Pós-Graduação em Enfermagem, Maringá, PR, Brazil; 2 Universidade Federal de São Paulo, Escola Paulista de Enfermagem, São Paulo, SP, Brazil; 3 Organização Pan-Americana da Saúde, Departamento de Doenças Transmissíveis e Determinantes Ambientais da Saúde, Brasília, DF, Brazil

**Keywords:** HIV, Tuberculosis, Coinfection, Time Series Studies, Regression Analysis, VIH, Tuberculosis, Coinfección, Estudios de Series Temporales, Análisis de Regresión, HIV, Tuberculose, Coinfecção, Estudos de Séries Temporais, Análise de Regressão

## Abstract

**Objective:**

To analyze the temporal trend in the incidence of tuberculosis-HIV coinfection in Brazil, by macro-region, Federative Unit, sex and age group, from 2010 to 2021.

**Methods:**

This was a time series study using surveillance data to estimate average annual percentage changes (AAPC), and 95% confidence intervals (95%CI) via joinpoint regression.

**Results:**

122,211 cases of tuberculosis-HIV coinfection were analyzed; a falling trend was identified for Brazil as a whole (AAPC = -4.3; 95%CI -5.1;-3.7), and in the country’s Southern (AAPC = -6.2; 95%CI -6.9;-5.5) and Southeast (AAPC = -4.6; 95%CI -5.6;-3.8) regions, even more so during the COVID-19 pandemic (2020-2021); the greatest falling trend was seen in Santa Catarina (AAPC = -9.3; 95%CI -10.1;-8.5), while the greatest rising trend was found in Tocantins (AAPC = 4.1; 95%CI 0.1;8.6); there was a rising trend among males, especially in Sergipe (AAPC = 3.9; 95%CI 0.4;7.9), and those aged 18 to 34 years, especially in Amapá (AAPC = 7.9; 95%CI 5.1;11.5).

**Conclusion:**

The burden and trends of tuberculosis-HIV coinfection were geographically and demographically disparate.

## RESUMEN

**Objetivo:**

Analizar la tendencia temporal de la incidencia de la coinfección tuberculosis-VIH en Brasil, por Macrorregión, Unidad Federativa, sexo y grupo de edad, 2010-2021.

**Métodos:**

Estudio de series de tiempo, con datos de vigilancia para la estimación de cambios porcentuales anuales promedio (CPAP) e intervalos de confianza del 95% (IC_95_%) vía joinpoint regression.

**Resultados:**

Se analizaron 122.211 casos de coinfección tuberculosis-VIH; se identificó tendencia decreciente en Brasil (CPAP = -4,3; IC_95_% -5,1;-3,7) y en las regiones Sur (CPAP = -6,2; IC_95_% -6,9;-5,5) y Sudeste (CPAP = -4,6; IC_95_% -5,6;-3,8), aumentando durante la pandemia de covid-19; mayor tendencia decreciente ocurrió en Santa Catarina (CPAP = -9,3; IC_95_% -10,1;-8,5) y creciente en Tocantins (CPAP = 4,1; IC_95_% 0,1;8,6); hubo tendencia al aumento en el sexo masculino, especialmente Sergipe (CPAP = 3,9; IC_95_% 0,4;7,9), y en los de 18 a 34 años, especialmente Amapá (CPAP = 7,9; IC_95_% 5,1;11,5).

**Conclusión:**

Había disparidades territoriales y demográficas en la carga y las tendencias de la coinfección tuberculosis-VIH.

## RESUMO

**Objetivo:**

Analisar a tendência temporal da incidência da coinfecção tuberculose-HIV no Brasil, por macrorregião, Unidade da Federação, sexo e faixa etária, 2010-2021.

**Métodos:**

Estudo de séries temporais, com dados de vigilância, para a estimativa de variações percentuais anuais médias (VPAM) e intervalos de confiança de 95% (IC_95_%), por *joinpoint regression*.

**Resultados:**

Foram analisados 122.211 casos de coinfecção tuberculose-HIV; identificou-se tendência decrescente no país (VPAM = -4,3; IC_95_% 5,1;-3,7) e em suas regiões Sul (VPAM = -6,2; IC_95_% -6,9;-5,5) e Sudeste (VPAM = -4,6; IC_95_% -5,6;-3,8), acentuada durante a pandemia de covid-19 (2020-2021); observou-se maior tendência decrescente em Santa Catarina (VPAM = -9,3; IC_95_% -10,1;-8,5) e maior tendência crescente no Tocantins (VPAM = 4,1; IC_95_% 0,1;8,6); houve tendência de incremento no sexo masculino, destacando-se Sergipe (VPAM = 3,9; IC_95_% 0,4;7,9), e na faixa etária de 18-34 anos, sobressaindo-se o Amapá (VPAM = 7,9; IC_95_% 5,1;11,5).

**Conclusão:**

Verificaram-se disparidades territoriais e demográficas na carga e nas tendências da coinfecção tuberculose-HIV.

## INTRODUCTION 

Tuberculosis (TB) and human immunodeficiency virus (HIV) infection overburden health systems, especially in countries with less availability of economic, human and structural resources.^1^ International agreements, expressed through the United Nations Sustainable Development Goals (SDGs), have been established to end the HIV and TB transmission – and therefore, TB-HIV coinfection as public health problem by 2030.^1,^ ^2^

It is estimated that a quarter of the world’s population is infected with TB. These are cases of infection that, eventually, can progress to the disease itself.^3^ Furthermore, TB persists as one of the main infectious causes of mortality in the global population, especially among those living with HIV.^3^ These people, compared to those not infected with HIV, have a high risk, up to 20 times greater, of progression from TB-infection to TB-disease, in addition to being more susceptible to unfavorable TB outcomes, such as death.^ 1,^ ^4^

The World Health Organization (WHO) compiled a list of 30 countries with the highest burden of TB-HIV coinfection. Developing countries and those with the largest populations, including Brazil, stood out on that list.^3^ Worldwide, in 2021, of the 6.4 million TB cases registered, 6.7% were people living with HIV and death was the outcome for 187,000 of them.^3^ In Brazil, the proportion of TB-HIV coinfection was 10.3% in 2019, with variations between national macro-regions and states.^5^

 Studies indicate that (i) individual factors, such as age, sex and degree of immunosuppression, (ii) socioeconomic factors, such as education and income, and (iii) programmatic factors, related to the organization of and access to health services, can increase the risk of TB in people with HIV.^6^ ^-9^ Furthermore, the absence and/or non-adoption of resources for prevention, diagnosis and treatment, both at the individual and programmatic level, may be related to higher incidence of dual infection.^6^ ^,7^

In this sense, the layout and regionalization of the health care network and its socio-spatial, economic and political inequalities must be considered. In the North, Midwest and Northeast regions of Brazil, services are concentrated in state capitals and their metropolitan regions, which can make access difficult for people living in peripheral areas.^10^ ^-12^ In the South and Southeast regions, the health care network is better distributed within the states, and health services, in general, have better performance.^10^ ^-12^

It is necessary to consider the way in which different contexts influence the incidence of TB-HIV coinfection, especially regarding infections with social, biological and environmental determinants. Time series studies, which consider territories and population strata, can be useful for Brazilian public health, since, based on the description of trends, it is possible to evaluate, direct and/or implement intervention strategies and policies.^6^

Brazil is a country with a high TB-HIV coinfection burden, with regional inequalities in the health care network and social and individual particularities that imply the possibility of dissimilarity in the incidence of these infections. Given these characteristics, it is necessary to identify the different behaviors of this condition in the country. In this sense, the objective of this study was to analyze the temporal trend in the incidence of TB-HIV coinfection in Brazil, by Macro-region, Federative Unit (FU), sex and age group, from 2010 to 2021.

## METHODS

This was an ecological study of time series of TB-HIV coinfection incidence in Brazil, by macro-region (North, Northeast, Midwest, South and Southeast) and FU, based on data from the Mortality Information System (*Sistema de Informações sobre Mortalidade* - SIM), the Notifiable Health Conditions Information System (*Sistema de Informação de Agravos de Notificação* - SINAN), the Medication Logistics Control System (*Sistema de Controle Logístico de Medicamentos* - SICLOM) and the National CD4+/CD8+ Lymphocyte Count and HIV Viral Load Network Laboratory Test Control System (Sistema de Controle de Exames Laboratoriais da Rede Nacional de Contagem de Linfócitos CD4+/CD8+ e Carga Viral do HIV - SISCEL).

The database was made available via the Fala.BR platform, on November 3, 2022, by the Department of HIV/AIDS, Tuberculosis, Viral Hepatitis and Sexually Transmitted Infections (*Departamento de HIV/Aids, Tuberculose, Hepatites Virais e Infecções Sexualmente Transmissíveis* - DATHI), located within the Health and Environment Surveillance Secretariat of the Ministry of Health: protocol number 25072.039887/2022-27. Probabilistic linkage of the systems (SIM, SINAN, SICLOM and SISCEL) was performed by the DATHI, as per the process described in the Epidemiological Bulletin – Epidemiological panorama of TB-HIV coinfection in Brazil, 2020 (*Boletim Epidemiológico – Panorama epidemiológico da coinfecção TB-HIV no Brasil, 2020*).^5^

Population data were obtained from the Brazilian National Health System Information Technology Department (*Departamento de Informática do Sistema Único de Saúde* - DATASUS) on November 4, 2022. With regard to the year 2010, we used the population data from the demographic census carried out by the Brazilian Institute of Geography and Statistics (*Instituto Brasileiro de Geografia e Estatística* - IBGE) that year; with regard to the intercensal years (2011-2021), we used population estimates prepared by the Health Ministry’s Department of Epidemiological Analysis and Surveillance of Noncommunicable Diseases (*Departamento de Análise Epidemiológica e Vigilância de Doenças Não Transmissíveis* - DAENT).^13^

The study population consisted of new cases reported on the SINAN-TB system, regardless of clinical form, with the “HIV” variable coded as “positive” or the “AIDS” (acquired immune deficiency syndrome) variable coded as as “yes”; or TB cases notified on the TB databases without one of these variables having been filled out, but for whom diagnosis had been recorded on the HIV databases, or who had a laboratory result on the SISCEL, or who had antiretroviral medication dispensation recorded on the Siclom.^5^

Cases from 2010 to 2021 were included in the study, considering DATHI availability of data on people aged 18 to 59, given that this age group corresponds to the majority of cases of TB-HIV coinfection (± 91.9%); children, adolescents and elderly people were not included, as they have particularities that would make it impossible to understand these specificities. Twelve records with the “sex” variable not filled out were excluded, given that this was one of the variables analyzed by this study.

Initially, we obtained crude incidence coefficients, year by year, by dividing the total number of new cases of TB-HIV coinfection by the resident population, in the same period and location; and multiplying the result by 100,000 inhabitants. After exploratory analysis of the data, we decided to calculate the incidence coefficients by sex (male; female) and age group (in years: 18-34; 35-59), taking the denominator as the population with the same demographic characteristics.

Trend analysis was performed using joinpoint regression, which allows checking whether straight segments would better explain the series than a single straight line. Given that twelve points were analyzed (one point for each year), we defined a maximum of two joinpoints, as established in the literature.^14^ The overall and stratified trend (sex and age group) was estimated for each macro-region and FU, assuming the influence of individual and programmatic aspects on the epidemiology of infections.

The annual incidence coefficients of TB-HIV coinfection, transformed by a natural logarithmic function (ln) due to better interpretation and comparison of results, were taken as the dependent variable (y); while the calendar years of the period were taken as the independent variable (x). The log-linear models [ln(y) = x’beta + error] were adjusted by the standard errors of the incidence coefficients and by correcting first-order autocorrelation, verified based on the data.^14^

The final models, estimated via grid search, were chosen by the lowest value of the weighted Bayesian information criterion. For each final model, we used the quantile-empirical method to calculate, (i) annual percentage change (APC), referring to the change in the values ​​of the incidence coefficients at each joinpoint, (ii) average annual percentage change (AAPC), relative to the geometric averages of the APCs, and (iii) the 95% confidence intervals (95%CI) of the APCs/AAPCs.^14^

When interpreting the calculated values, positive APCs/AAPCs indicated a rising trend in TB-HIV coinfection incidence coefficients, while negative APCs/AAPCs indicated a falling trend. APCs/AAPCs values with 95%CIs that did not include the null value (zero) were considered to be significant. Non-significant changes were interpreted as having a stationary trend. The analyses were performed using version 5.0.2. of the Joinpoint Regression Program®.^14^

In accordance with National Health Council Resolution No. 466, dated December 12, 2012, the study was approved by the *Universidade Estadual de Maringá* Research Ethics Committee, as per Opinion No. 5.721.740, issued on October 25, 2022: Certificate of Submission for Ethical Appraisal (*Certificado de Apresentação para Apreciação Ética* - CAAE) No. 63981922.6.0000.0104.

## RESULTS

**Figure 1:**
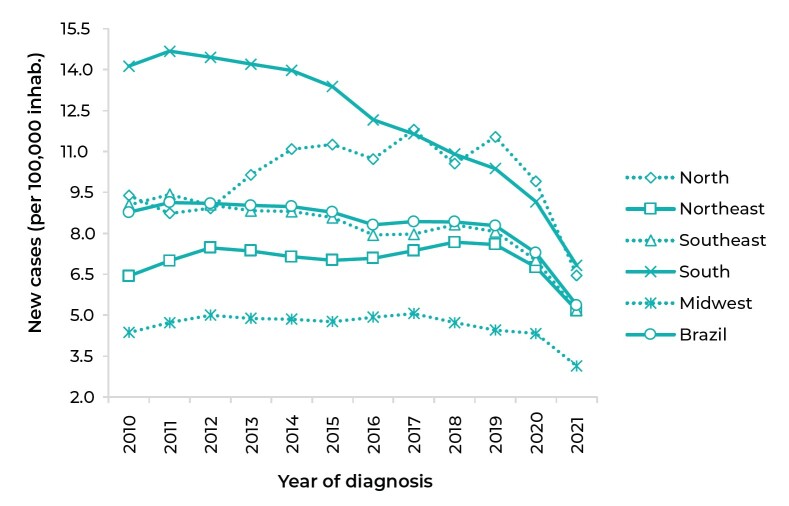
Time series of crude tuberculosis-HIV coinfection incidence coefficients (per 100,000 inhab.) in the population aged 18-59 years, by macro-region, Brazil, 2010-2021

We analyzed 122,211 new cases of TB-HIV coinfection notified between 2010 and 2021 in the population aged 18 to 59 years in Brazil. The annual incidence coefficients of TB-HIV coinfection in the period, for each national macro-region and for the country as a whole, are shown in Figure 1. Table 1 presents the crude incidence coefficients of dual infection, year by year, by FU.

**Table 1:** Crude incidence coeffcients for tuberculosis-HIV coinfection (per 100,000 inhab.) in the population aged 18-59 years, by Federative Unit, Brazil, 2010-2021

Federative Units (by macro-region)		2010	2011	2012	2013	2014	2015	2016	2017	2018	2019	2020	2021
**North**	Rondônia	4.9	7.2	6.8	7.7	8.6	6.9	9.6	8.0	6.2	6.9	5.4	4.1
	Acre	4.1	3.7	4.5	3.0	3.0	2.4	2.2	2.7	2.3	4.2	1.8	2.7
	Amazonas	18.6	16.0	17.4	21.5	25.3	25.9	23.1	24.6	21.8	21.9	18.6	12.7
	Roraima	8.9	5.4	6.4	9.1	3.9	7.5	5.3	7.2	5.5	7.3	8.5	7.5
	Pará	8.7	8.3	7.9	8.5	8.6	9.0	8.6	10.3	9.4	11.0	9.7	5.1
	Amapá	3.3	3.6	4.7	2.9	4.2	3.6	4.2	5.5	5.4	6.2	4.3	5.0
	Tocantins	2.0	1.2	1.2	1.4	2.4	1.5	1.9	1.6	2.3	1.9	1.8	2.9
**Northeast**	Maranhão	5.0	4.8	5.5	5.8	5.1	5.7	5.6	5.9	7.0	6.8	6.0	4.8
	Piauí	3.2	3.8	4.0	2.6	4.1	2.4	3.6	3.6	4.1	3.7	3.1	2.5
	Ceará	5.3	6.8	7.9	6.8	6.9	7.5	6.8	7.6	7.6	8.6	7.4	5.6
	Rio Grande do Norte	5.3	6.2	8.2	7.5	7.1	7.1	5.3	7.2	8.5	8.1	7.6	5.0
	Paraíba	5.8	5.5	5.5	6.6	6.2	5.6	4.4	5.9	6.4	6.0	5.9	4.8
	Pernambuco	12.2	13.4	14.8	13.8	13.6	13.6	14.2	13.8	13.6	12.7	11.6	8.7
	Alagoas	6.0	6.6	8.2	8.8	7.3	5.9	9.5	9.1	9.1	9.6	7.6	5.2
	Sergipe	3.7	4.1	3.3	4.7	4.4	3.4	4.2	4.4	6.7	5.4	5.9	3.8
	Bahia	5.7	5.8	5.1	5.6	5.5	5.3	5.2	5.1	5.0	5.1	4.4	3.7
**Southeast**	Minas Gerais	3.4	4.1	3.9	4.1	4.1	3.6	3.3	3.2	3.3	3.5	3.0	1.9
	Espírito Santo	5.7	5.2	6.1	5.7	5.1	5.6	4.3	4.4	5.5	4.9	6.4	6.7
	Rio de Janeiro	15.9	16.9	16.6	15.0	15.4	15.8	14.8	14.6	15.2	14.8	12.8	9.5
	São Paulo	9.3	9.4	8.8	9.0	8.8	8.4	7.8	8.0	8.3	7.9	6.8	5.0
**South**	Paraná	6.0	5.4	5.4	5.6	5.5	5.3	4.7	4.3	4.5	3.7	3.9	3.2
	Santa Catarina	12.3	14.1	11.9	11.9	11.9	11.0	9.8	10.0	8.9	8.3	6.1	4.6
	Rio Grande do Sul	23.1	24.2	25.0	24.2	23.8	22.9	21.2	20.1	18.8	18.6	16.6	12.0
**Midwest**	Mato Grosso do Sul	6.6	7.4	8.8	8.8	7.9	6.6	6.6	6.8	8.0	9.6	9.3	6.5
	Mato Grosso	7.1	6.9	5.2	6.8	6.5	6.7	8.8	8.0	6.8	6.0	5.3	3.8
	Goiás	3.1	3.3	4.1	3.3	3.1	3.9	3.2	3.8	3.3	2.3	2.6	2.0
	Distrito Federal	2.1	3.1	3.5	3.0	4.2	2.9	3.1	3.2	2.9	3.2	2.8	2.1

We identified a falling trend in TB-HIV coinfection incidence in Brazil as a whole: AAPC = -4.3; 95%CI -5.1;-3.7. In the Northern region (APC = 3.1; 95%CI 1.0;6.8) and the Northeast region (APC = 1.3; 95%CI 0.2;3.0) coefficients showed a rising trend between 2010 and 2019. In the Southern region (AAPC = -6.2; 95%CI -6.9;5.5) and Southeast region (AAPC = -4.6; 95%CI -5.6;-3.8) there was a falling trend throughout the entire study period, from 2010 to 2021. All regions showed a drop in coefficients between 2019 and 2021 (Table 2).

**Table 2:** Temporal trend of crude incidence coefficients for tuberculosis-HIV coinfection (per 100,000 inhab.) in the population aged 18-59 years, by macro-region and Federative Unit, Brazil, 2010-2021

Macro-regions and Federative Units	Period	APC^a^ (95%CI^b^)	AAPCc (95%CI^b^)
North	2010-2019	3.1 (1.0;6.8)^d^	-2.4 (-7.1;0.1)
	2019-2021	-23.7 (-64.3;-8.4)^d^	
Rondônia	2010-2016	6.7 (1.4;17.4)^d^	-2.4 (-7.1;0.7)
	2016-2021	-12.4 (-29.1;-7.2)^d^	
Acre	2010-2016	-11.2 (-17.4;-8.3)^d^	-7.1 (-10.0;-5.0)^d^
	2016-2019	13.4 (-6.0;22.1)	
	2019-2021	-21.1 (-34.9;0.2)	
Amazonas	2010-2015	9.5 (-6.4;37.4)	-1.7 (-4.7;2.1)
	2015-2019	-3.8 (-9.2;21.9)	
	2019-2021	-21.6 (-36.2;-8.0)^d^	
Roraima	2010-2017	-2.4 (-16.9;1.9)	1.5 (-1.2;3.9)
	2017-2021	8.8 (0.6;29.6)^d^	
Pará	2010-2019	3.4 (2.0;5.9)^d^	-1.6 (-3.4;0.6)
	2019-2021	-21.3 (-30.3;-7.4)^d^	
Amapá	2010-2015	0.0 (-15.0;5.2)	1.7 (-1.3;3.6)
	2015-2018	17.0 (7.9;25.7)^d^	
	2018-2021	-9.1 (-26.1;-1.9)^d^	
Tocantins	2010-2021	4.1 (0.1;8.6)^d^	4.1 (0.1;8.6)^d^
**Northeast**	2010-2019	1.3 (0.2;3.0)^d^	-2.2 (-3.5;-1.0)^d^
	2019-2021	-16.8 (-22.9;-8.7)^d^	
Maranhão	2010-2016	2.1 (-4.1;4.3)	-0.4 (-1.7;0.7)
	2016-2019	7.6 (3.9;10.8)^d^	
	2019-2021	-17.5 (-23.5;-9.2)^d^	
Piauí	2010-2015	-4.6 (-13.5;-1.5)^d^	-3.4 (-6.1;-2.2)^d^
	2015-2018	11.1 (2.8;17.4)^d^	
	2018-2021	-14.4 (-30.9;-7.8)^d^	
Ceará	2010-2019	3.1 (1.1;13.2)^d^	-0.7 (-3.2;2.6)
	2019-2021	-16.2 (-28.7;-0.8)^d^	
Rio Grande do Norte	2010-2021	0.7 (-3.3;5.4)	0.7 (-3.3;5.4)
Paraíba	2010-2021	-0.4 (-2.6;1.9)	-0.4 (-2.6;1.9)
Pernambuco	2010-2012	7.3 (1.0;16.1)^d^	-2.8 (-4.2;-1.4)^d^
	2012-2018	-1.0 (-2.6;0.3)	
	2018-2020	-17.5 (-23.8;-11.1)^d^	
Alagoas	2010-2019	4.3 (1.8;11.6)^d^	-1.8 (-5.3;2.3)
	2019-2021	-25.3 (-40.2;-5.1)^d^	
Sergipe	2010-2021	4.0 (1.2;7.2)^d^	4.0 (1.2;7.2)^d^
Bahia	2010-2019	-1.4 (-1.8;-0.8)^d^	-3.6 (-4.2;-3.1)^d^
	2019-2021	-13.1 (-16.2;-8.2)^d^	
**Southeast**	2010-2019	-1.7 (-2.4;-0.7)^d^	-4.6 (-5.6;-3.8)^d^
	2019-2021	-16.9 (-21.9;-10.1)^d^	
Minas Gerais	2010-2019	-1.5 (-7.7;24.0)	-5.8 (-10.1;-0.3)^d^
	2019-2021	-22.8 (-42.4;-0.8)^d^	
Espírito Santo	2010-2013	2.0 (-1.8;12.0)	1.9 (0.9;3.3)^d^
	2013-2017	-6.5 (-11.3;-3.2)^d^	
	2017-2021	11.1 (6.7;19.1)^d^	
Rio de Janeiro	2010-2019	-1.2 (-1.9;-0.2)^d^	-4.4 (-5.5;-3.6)^d^
	2019-2021	-17.6 (-23.0;-10.6)^d^	
São Paulo	2010-2016	-2.7 (-5.9;-1.6)^d^	-5.4 (-6.2;-4.8)^d^
	2016-2019	0.2 (-1.9;2.0)	
	2019-2021	-20.3 (-24.2;-14.3)^d^	
**South**	2010-2014	-0.9 (-2.3;2.4)	-6.2 (-6.9;-5.5)^d^
	2014-2019	-5.8 (-7.1;-4.4)^d^	
	2019-2021	-16.5 (-20.5;-12.2)^d^	
Paraná	2010-2014	-0.5 (-3.0;5.0)	-4.4 (-5.4;-3.4)^d^
	2014-2021	-6.5 (-9.1;-5.4)^d^	
Santa Catarina	2010-2014	-3.2 (-4.7;0.6)	-9.3 (-10.1;-8.5)^d^
	2014-2019	-6.5 (-8.6;-5.1)^d^	
	2019-2021	-26.1 (-29.9;-19.5)^d^	
Rio Grande do Sul	2010-2012	5.0 (-0.4;10.2)	-5.2 (-6.3;-4.4)^d^
	2012-2019	-4.3 (-5.4;-3.4)^d^	
	2019-2021	-17.3 (-22.1;-11.7)^d^	
**Midwest**	2010-2018	0.7 (-0.4;2.1)	-2.6 (-3.9;-1.7)^d^
	2018-2021	-10.9 (-20.2;-6.5)^d^	
Mato Grosso do Sul	2010-2021	0.7 (-4.7;6.9)	0.7 (-4.7;6.9)
Mato Grosso	2010-2012	-12.0 (-19.7;0.4)	-5.1 (-6.9;-3.3)^d^
	2012-2017	8.0 (4.2;16.7)^d^	
	2017-2021	-16.1 (-23.4;-11.3)^d^	
Goiás	2010-2017	0.8 (-2.2;15.7)	-4.5 (-8.6;-0.6)^d^
	2017-2021	-13.0 (-36.1;-6.0)^d^	
Distrito Federal	2010-2012	26.7 (4.4;48.3)^d^	1.4 (-1.4;4.0)
	2012-2021	-3.5 (-7.1;-1.9)^d^	
**B** **razil**	2010-2019	-1.0 (-1.6;-0.2)^d^	-4.3 (-5.1;-3.7)^d^
	2019-2021	-18.0 (-22.1;-11.7)^d^	

a) APC: Annual percentage change; b) 95%CI: 95% confidence interval (lower limit; upper limit); c) AAPC: Average annual percentage change; d) Statistically significant value.

The analysis by FU showed a rising trend in TB-HIV coinfection incidence in Tocantins, Sergipe and Espírito Santo, throughout the time series. The states of Acre, Piauí, Pernambuco, Bahia, Minas Gerais, Rio de Janeiro, São Paulo, Paraná, Santa Catarina, Rio Grande do Sul, Mato Grosso and Goiás had fallings trends, from 2010 to 2021. The majority of FU (14; 51.8%) recorded a drop in TB-HIV coinfection incidence with effect from 2018 or 2019 (Table 2).

A falling trend was identified in the coefficients among the female population, in Brazil as a whole and its macro-regions, from 2010 to 2020. A greater increase in trends was seen for Maranhão (AAPC = 3.3; 95%CI 1.3;5 .6) and greater decrease for Acre (AAPC = -15.8; 95%CI -27.6;-9.0). Some FU (seven) registered positive APCs in incidence among the female population, such as Acre, Amazonas, Bahia and Espírito Santo (Table 3).

**Table 3:** Temporal trend of crude incidence coefficients for tuberculosis-HIV coinfection (per 100,000 inhab.) in the population aged 18-59 years, by sex (male; female), by macro-region and Federative Unit, Brazil, 2010-2021

Macro-regions and Federative Units	**Female**			Male		
	**Period**	**APC** ^a^ **(95%CI** ^b^ **)**	**AAPC** ^c^ **(95%CI** ^b^ **)**	**Period**	**APC** ^a^ **(95%CI** ^b^ **)**	**AAPC** ^c^ **(95%CI** ^b^ **)**
North	2010-2019	1.3 (-0.9;7.7)	-3.5 (-8.4;0.0)	2010-2014	7.2 (3.2;37.2)^d^	-0.2 (-2.7;2.0)
	2019-2021	-22.5 (-64.0;-4.6)^d^		2014-2019	1.5 (-3.2;5.2)	
	–	–	–	2019-2021	-17.4 (-44.3;-10.2)^d^	
Rondônia	2010-2018	1.8 (-2.2;11.8)	-6.6 (-13.0;-2.0)^d^	2010-2015	11.7 (3.3;47.7)^d^	-0.8 (-5.8;5.4)
	2018-2021	-25.8 (-55.4;-9.6)^d^		2015-2021	-10.1 (-25.9;-5.0)^d^	
Acre	2010-2013	-44.3 (-72.9;-21.4)^d^	-15.8 (-27.6;-9.0)^d^	2010-2021	-2.9 (-7.7;1.6)	-2.9 (-7.7;1.6) – –
	2013-2016	34.2 (0.1;74.0)^d^		–	–	
	2016-2021	-18.4 (-66.8;-2.1)^d^		–	–	
Amazonas	2010-2017	4.1 (0.4;14.4)^d^	-2.8 (-7.0;0.9)	2010-2015	11.7 (5.6;31.3)^d^	1.8 (-1.2;5.8)
	2017-2021	-13.8 (-35.0;-6.1)^d^		2015-2021	-5.8 (-14.0;-1.9)^d^	
Roraima	2010-2021	-5.1 (-11.4;1.3)	-5.1 (-11.4;1.3)	2010-2021	1.8 (-4.1;9.0)	1.8 (-4.1;9.0)
Pará	2010-2021	-0.1 (-2.7;2.7)	-0.1 (-2.7;2.7) – –	2010-2016	1.4 (-4.8;3.2)	-2.6 (-4.2;-1.5)^d^
	–	–		2016-2019	10.4 (5.5;14.4)^d^	
	–	–		2019-2021	-28.6 (-35.1;-22.2)^d^	
Amapá	2010-2021	-0.9 (-8.5;8.3)	-0.9 (-8.5;8.3)	2010-2018	8.6 (6.5;22.4)^d^	5.6 (3.3;8.7)^d^
	–	–	–	2018-2021	-2.2 (-16.6;5.4)	
Tocantins	2010-2021	3.0 (-6.6;15.4)	3.0 (-6.6;15.4)	2010-2021	4.2 (-3.3;13.4)	4.2 (-3.3;13.4)
**Northeast**	2010-2019	0.7 (-0.7;3.7)	-3.3 (-5.6;-1.3)^d^	2010-2019	1.5 (0.6;3.0)^d^	-1.5 (-2.6;-0.4)^d^
	2019-2021	-19.6 (-31.2;-7.5)^d^		2019-2021	-13.9 (-19.8;-6.4)^d^	
Maranhão	2010-2021	3.3 (1.3;5.6)^d^	3.3 (1.3;5.6)^d^	2010-2021	2.5 (1.3;3.7)^d^	2.5 (1.3;3.7)^d^
Piauí	2010-2018	0.4 (-4.1;40.8)	-5.4 (-11.4;1.7)	2010-2015	-4.8 (-12.4;-1.8)^d^	-3.7 (-5.9;-1.9)^d^
	2018-2021	-19.1 (-48.7;-4.1)^d^		2015-2019	9.7 (4.4;19.5)^d^	
	–	–	–	2019-2021	-23.3 (-34.3;-9.9)^d^	
Ceará	2010-2012	15.8 (5.6;27.3)^d^	-1.3 (-3.3;0.5)	2010-2021	1.0 (-1.8;4.2)	1.0 (-1.8;4.2)
	2012-2019	2.4 (-0.4;4.4)		–	–	–
	2019-2021	-26.1 (-33.3;-15.8)^d^		–	–	–
Rio Grande do Norte	2010-2021	-2.0 (-8.8;5.4)	-2.0 (-8.8;5.4)	2010-2021	1.4 (-1.3;4.6)	1.4 (-1.3;4.6)
Paraíba	2010-2021	-2.8 (-7.1;1.5)	-2.8 (-7.1;1.5)	2010-2021	0.5 (-1.8;3.0)	0.5 (-1.8;3.0)
Pernambuco	2010-2012	9.3 (1.6;16.5)^d^	-3.4 (-4.9;-2.1)	2010-2018	1.3 (-0.4;4.3)	-2.7 (-4.5;-1.1)^d^
	2012-2019	-2.3 (-4.1;-0.9)^d^		2018-2021	-12.6 (-22.7;-6.1)^d^	
	2019-2021	-17.9 (-24.3;-9.9)^d^		–	–	–
Alagoas	2010-2019	3.0 (-8.9;55.6)	-3.0 (-9.2;7.1)	2010-2018	6.2 (3.1;14.6)^d^	-0.2 (-3.8;3.5)
	2019-2021	-25.8 (-51.4;5.8)		2018-2021	-15.4 (-35.2;-4.3)^d^	
Sergipe	2010-2021	3.3 (-2.1;9.9)	3.3 (-2.1;9.9)	2010-2021	3.9 (0.4;7.9)^d^	3.9 (0.4;7.9)^d^
Bahia	2010-2012	-14.2 (-17.5;-7.2)^d^	-5.3 (-6.3;-4.3)^d^	2010-2013	1.3 (0.1;4.1)^d^	-3.1 (-3.5;-2.8)^d^
	2012-2019	1.2 (0.3;3.5)^d^		2013-2019	-2.5 (-3.0;-1.8)^d^	
	2019-2021	-16.8 (-22.3;-9.9)^d^		2019-2021	-11.4 (-13.3;-7.9)^d^	
**Southeast**	2010-2019	-3.1 (-4.1;-0.2)^d^	-5.6 (-7.4;-3.9)^d^	2010-2019	-1.2 (-1.8;-0.5)^d^	-4.3 (-5.0;-3.6)^d^
	2019-2021	-16.5 (-25.4;-6.0)^d^		2019-2021	-16.8 (-20.9;-10.5)^d^	
Minas Gerais	2010-2012	11.9 (-10.0;45.2)	-8.1 (-14.0;-3.5)^d^	2010-2021	-2.4 (-4.8;0.0)	-2.4 (-4.8;0.0) – –
	2012-2019	-6.1 (-17.4;7.6)		–	–	
	2019-2021	-29.9 (-51.7;-4.3)^d^		–	–	
Espírito Santo	2010-2013	14.5 (9.4;28.9)^d^	2.6 (1.4;4.2)^d^	2010-2017	-3.2 (-6.0;-1.5)^d^	1.2 (0.0;2.3)
	2013-2016	-17.3 (-20.9;-10.3)^d^		2017-2021	9.5 (4.7;19.2)^d^	
	2016-2021	9.4 (5.9;16.0)^d^		–	–	–
Rio de Janeiro	2010-2019	-2.7 (-6.4;8.5)	-4.6 (-6.7;-2.0)^d^	2010-2019	-0.7 (-1.3;0.1)	-4.3 (-5.4;-3.7)^d^
	2019-2021	-12.8 (-23.8;-2.1)^d^		2019-2021	-19.2 (-24.3;-12.1)^d^	
São Paulo	2010-2016	-4.6 (-7.5;-3.8)^d^	-6.7 (-7.6;-6.1)^d^	2010-2019	-1.5 (-2.2;-0.6)^d^	-4.8 (-5.8;-4.0)^d^ –
	2016-2019	0.6 (-2.2;2.5)		2019-2021	-18.4 (-23.5;-11.2)^d^	
	2019-2021	-21.9 (-26.4;-16.6)^d^		–	–	
**South**	2010-2013	3.2 (0.8;6.9)^d^	-5.8 (-6.6;-5.2)^d^	2010-2014	-1.7 (-4.7;5.9)	-6.3 (-7.5;-5.2)^d^
	2013-2019	-5.4 (-6.3;-4.4)^d^		2014-2019	-5.8 (-7.5;-2.3)^d^	
	2019-2021	-18.8 (-22.6;-13.8)^d^		2019-2021	-15.9 (-22.4;-9.5)^d^	
Paraná	2010-2015	1.4 (-1.5;6.3)	-6.2 (-7.8;-5.0)^d^	2010-2014	-2.0 (-3.9;4.4)	-4.0 (-5.3;-3.1)^d^
	2015-2021	-12.2 (-16.2;-10.0)^d^		2014-2021	-5.1 (-12.2;-4.2)^d^	
Santa Catarina	2010-2016	-4.3 (-7.4;2.2)	-10.0 (-11.6;-8.9)^d^	2010-2018	-4.4 (-5.8;-2.7)^d^	-8.7 (-10.7;-7.4)^d^ –
	2016-2019	-8.0 (-10.3;-3.0)^d^		2018-2021	-19.0 (-31.9;-13.0)^d^	
	2019-2021	-27.7 (-35.4;-20.1)^d^		–	–	
Rio Grande do Sul	2010-2013	4.9 (4.0;5.7)^d^	-4.3 (-4.7;-3.9)^d^	2010-2012	3.6 (-5.6;15.4)	-5.6 (-7.8;-3.7)^d^
	2013-2019	-4.7 (-5.1;-4.2)^d^		2012-2019	-4.6 (-7.7;0.1)	
	2019-2021	-15.5 (-17.6;-11.3)^d^		2019-2021	-17.1 (-27.2;-7.7)^d^	
**Midwest**	2010-2016	0.6 (-1.3;3.5)	-3.9 (-5.4;-3.0)^d^	2010-2014	0.9 (-3.4;2.6)	-3.5 (-4.3;-3.0)^d^
	2016-2021	-9.2 (-14.5;-6.6)^d^		2014-2017	5.8 (2.9;7.9)^d^	
	–	–	–	2017-2021	-13.9 (-16.2;-12.4)^d^	
Mato Grosso do Sul	2010-2021	-0.6 (-5.8;4.9)	-0.6 (-5.8;4.9)	2010-2021	1.3 (-3.0;6.3)	1.3 (-3.0;6.3)
Mato Grosso	2010-2017	0.1 (-3.7;19.2)	-4.5 (-9.2;-0.7)^d^	2010-2013	-7.0 (-19.7;0.2)	-4.6 (-6.8;-3.2)^d^
	2017-2021	-12.2 (-37.5;-4.3)^d^		2013-2017	12.8 (6.6;22.6)^d^	
	–	–	–	2017-2021	-17.9 (-26.3;-12.7)^d^	
Goiás	2010-2017	1.3 (-1.6;6.7)	-4.9 (-7.8;-2.7)^d^	2010-2017	0.6 (-2.4;21.1)	-4.4 (-8.6;-0.1)^d^
	2017-2021	-14.8 (-30.8;-8.4)^d^		2017-2021	-12.5 (-36.2;-5.5)^d^	
Distrito Federal	2010-2018	-8.2 (-25.0;5.4)	-3.3 (-8.0;-0.2)^d^	2010-2012	29.9 (11.4;48.5)^d^	0.1 (-2.7;2.6)
	2018-2021	10.9 (-6.8;45.0)		2012-2019	-0.9 (-3.1;1.6)	
	–	–	–	2019-2021	-20.1 (-30.7;-8.4)^d^	
**B** **razil**	2010-2019	-1.8 (-2.6;-0.9)^d^	-5.4 (-6.5;-4.6)^d^	2010-2019	-0.7 (-1.2;-0.1)^d^	-4.0 (-4.6;-3.4)^d^
	2019-2021	-19.8 (-25.4;-12.9)^d^		2019-2021	-17.3 (-20.8;-11.2)^d^	

a) APC: Annual percentage change; b) 95%CI: 95% confidence interval (lower limit; upper limit); c) AAPC: Average annual percentage change; d) Statistically significant value.

As for the male population, there was a falling trend in incidence coefficients in Brazil as a whole and in most macro-regions; the exception was the Northern region, where the trend proved to be stable. The most pronounced rising and falling trends were, respectively, in Amapá (AAPC = 5.6; 95%CI 3.3;8.7) and Santa Catarina (AAPC = -8.7; 95%CI -10, 7;-7.4). Nine FU – including Pará, Rondônia, Amazonas, Piauí and Espírito Santo – registered positive APCs (Table 3).

TB-HIV coinfection incidence in the 18-34 age group showed a falling trend for Brazil as a whole, and also in the South, Southeast and Northeast macro-regions. Still with regard to this age group, a greater increase and a greater decline in TB-HIV incidence were seen, respectively, in Amapá (AAPC = 7.9; 95%CI 5.1;11.5) and in Santa Catarina (AAPC = - 9.7; 95%CI -12.0;-7.7). Furthermore, for the same age group, ten FU had positive APCs in segments of the series, such as Amazonas, Mato Grosso, Ceará, Alagoas and Rio Grande do Norte (Table 4).

**Table 4:** Temporal trend of crude incidence coefficients for tuberculosis-HIV coinfection (per 100,000 inhab.) in the population, by age group (18-34 years; 35-59 years), by macro-region and Federative Unit, Brazil, 2010-2021

Macro-regions and Federative Units	**18-34 years**			35-59 years		
	**Period**	**APC** ^a^ **(95%CI** ^b^ **)**	**AAPC** ^c^ **(95%CI** ^b^ **)**	**Period**	**APC** ^a^ **(95%CI** ^b^ **)**	**AAPC** ^c^ **(95%CI** ^b^ **)**
North	2010-2019	2.8 (0.3;9.1)^d^	-2.9 (-7.3;0.6)	2010-2014	7.2 (4.5;15.9)^d^	-2.5 (-3.6;-1.1)^d^
	2019-2021	-24.9 (-45.7;-6.4)^d^		2014-2019	0.1 (-2.5;2.6)	
	–	–	–	2019-2021	-24.7 (-30.0;-16.0)^d^	
Rondônia	2010-2016	5.7 (1.3;15.9)^d^	-2.6 (-6.3;0.4)	2010-2012	26.3 (10.0;43.5)^d^	-1.1 (-3.6;0.9)
	2016-2021	-11.6 (-26.3;-6.5)^d^		2012-2017	1.7 (-5.6;5.4)	
	–	–	–	2017-2021	-15.5 (-25.4;-11.3)^d^	
Acre	2010-2021	-4.9 (-9.1;-1.3)^d^	-4.9 (-9.1;-1.3)^d^	2010-2021	-3.6 (-10.5;3.1)	-3.6 (-10.5;3.1)
Amazonas	2010-2017	5.7 (1.3;40.1)^d^	-1.1 (-6.8;5.3)	2010-2015	11.1 (0.3;29.7)^d^	-1.6 (-4.0;1.3)
	2017-2021	-12.0 (-41.1;-2.5)^d^		2015-2019	-5.3 (-8.8;17.9)	
	–	–	–	2019-2021	-21.8 (-32.8;-10.1)^d^	
Roraima	2010-2021	5.2 (0.7;10.7)^d^	5.2 (0.7;10.7)^d^	2010-2013	16.0 (3.6;48.9)^d^	1.3 (-1.5;4.5)
	–	–	–	2013-2016	-25.0 (-32.4;-13.0)^d^	
	–	–	–	2016-2021	11.8 (4.0;33.3)^d^	
Pará	2010-2012	-10.3 (-16.2;0.3)	-3.6 (-5.4;-1.9)^d^	2010-2019	3.3 (1.7;6.4)^d^	-1.8 (-3.8;0.7)
	2012-2019	5.4 (4.0;11.3)^d^		2019-2021	-22.0 (-31.5;-6.8)^d^	
	2019-2021	-24.3 (-32.7;-13.3)^d^		–	–	–
Amapá	2010-2021	7.9 (5.1;11.5)^d^	7.9 (5.1;11.5)^d^	2010-2021	1.5 (-3.6;7.4)	1.5 (-3.6;7.4)
Tocantins	2010-2021	5.5 (-1.0;13.5)	5.5 (-1.0;13.5)	2010-2021	2.6 (-3.9;10.7)	2.6 (-3.9;10.7)
**Northeast**	2010-2019	1.6 (0.2;3.9)^d^	-2.5 (-4.5;-0.9)^d^	2010-2019	0.8 (-0.2;2.4)	-2.3 (-3.6;-1.2)^d^
	2019-2021	-19.1 (-28.0;-8.2)^d^		2019-2021	-15.3 (-21.8;-7.6)^d^	
Maranhão	2010-2019	2.2 (0.8;5.0)^d^	-1.7 (-3.6;0.4)	2010-2015	0.1 (-4.4;2.4)	-0.2 (-1.3;0.9)
	2019-2021	-17.2 (-27.2;-3.8)^d^		2015-2019	9.9 (6.9;15.1)^d^	
	–	–	–	2019-2021	-18.2 (-23.5;-10.8)^d^	
Piauí	2010-2019	2.4 (-1.3;10.2)	-8.1 (-15.4;-2.3)^d^	2010-2021	-0.6 (-3.1;1.7)	-0.6 (-3.1;1.7)
	2019-2021	-43.6 (-65.4;-10.7)^d^		–	–	–
Ceará	2010-2019	4.0 (1.4;12.9)^d^	-1.4 (-4.3;2.6)	2010-2019	2.1 (-0.3;17.9)	-0.6 (-2.7;3.1)
	2019-2021	-22.2 (-35.0;-2.9)^d^		2019-2021	-11.7 (-23.3;0.8)	
Rio Grande do Norte	2010-2019	3.1 (0.4;18.2)^d^	-3.1 (-7.3;2.6)	2010-2021	0.7 (-2.8;4.9)	0.7 (-2.8;4.9)
	2019-2021	-26.4 (-43.9;-2.7)^d^		–	–	–
Paraíba	2010-2019	1.7 (-0.4;10.5)	-2.7 (-5.4;0.8)	2010-2021	-0.6 (-2.4;1.3)	-0.6 (-2.4;1.3)
	2019-2021	-20.2 (-33.1;-2.9)^d^		–	–	–
Pernambuco	2010-2012	9.1 (3.0;16.9)^d^	-2.0 (-3.2;-0.8)^d^	2010-2018	0.9 (-0.5;3.1)	-3.6 (-5.6;-2.4)^d^
	2012-2019	-1.8 (-3.1;-0.5)^d^		2018-2021	-14.6 (-28.1;-9.0)^d^	
	2019-2021	-12.6 (-18.8;-6.9)^d^		–	–	–
Alagoas	2010-2019	6.2 (3.5;12.3)^d^	-1.7 (-5.7;2.3)	2010-2019	2.5 (-0.5;32.3)	-2.3 (-6.2;4.3)
	2019-2021	-30.7 (-46.4;-9.9)^d^		2019-2021	-21.4 (-39.5;-0.8)^d^	
Sergipe	2010-2021	4.2 (1.0;8.0)^d^	4.2 (1.0;8.0)^d^	2010-2016	-1.1 (-12.8;2.4)	-0.5 (-3.8;1.9)
	–	–	–	2016-2019	20.1 (9.2;29.9)^d^	
	–	–	–	2019-2021	-23.6 (-37.4;-6.8)	
Bahia	2010-2012	-3.8 (-5.4;-1.5)^d^	-4.5 (-5.0;-4.1)^d^	2010-2019	-2.1 (-2.6;-1.2)^d^	-3.8 (-4.5;-3.1)^d^
	2012-2019	-0.6 (-1.0;0.7)		2019-2021	-11.2 (-15.1;-5.9)^d^	
	2019-2021	-17.6 (-20.3;-15.6)^d^		–	–	–
**Southeast**	2010-2019	0.0 (-0.8;1.1)	-3.0 (-4.0;-2.1)^d^	2010-2019	-2.9 (-3.6;-1.8)^d^	-6.0 (-7.2;-5.1)^d^
	2019-2021	-15.3 (-20.6;-8.3)^d^		2019-2021	-18.7 (-24.7;-10.8)^d^	
Minas Gerais	2010-2019	-0.4 (-3.1;9.8)	-4.5 (-7.6;-0.9)^d^	2010-2019	-2.3 (-9.3;26.2)	-6.8 (-11.5;-0.6)^d^
	2019-2021	-20.9 (-34.4;-3.5)^d^		2019-2021	-24.4 (-45.5;-1.0)^d^	
Espírito Santo	2010-2012	21.8 (5.9;38.4)^d^	4.5 (2.4;6.7)^d^	2010-2017	-4.4 (-7.3;-2.3)^d^	0.6 (-0.7;1.9)
	2012-2018	-6.2 (-13.4;-4.1)^d^		2017-2021	10.1 (5.0;20.1)^d^	
	2018-2021	17.2 (6.7;36.2)^d^		–	–	–
Rio de Janeiro	2010-2019	-0.4 (-1.1;0.8)	-2.9 (-4.0;-2.1)^d^	2010-2019	-1.8 (-2.8;-0.3)^d^	-5.6 (-7.0;-4.3)^d^
	2019-2021	-13.6 (-19.3;-6.6)^d^		2019-2021	-20.8 (-28.1;-10.6)^d^	
São Paulo	2010-2016	-0.8 (-2.5;-0.2)^d^	-3.4 (-4.0;-2.8)^d^	2010-2019	-3.5 (-4.0;-2.8)^d^	-6.6 (-7.3;-5.9)^d^
	2016-2019	3.9 (1.6;5.6)^d^		2019-2021	-19.3 (-23.2;-12.9)^d^	
	2019-2021	-19.8 (-22.9;-16.5)^d^		–	–	–
**South**	2010-2012	0.0 (-3.1;2.5)	-6.9 (-7.5;-6.4)^d^	2010-2013	2.2 (-0.5;9.1)	-5.6 (-6.6;-4.8)^d^
	2012-2019	-6.0 (-6.8;-5.4)^d^		2013-2019	-5.0 (-6.1;-3.7)^d^	
	2019-2021	-16.2 (-18.9;-12.1)^d^		2019-2021	-18.0 (-22.6;-12.5)^d^	
Paraná	2010-2013	3.9 (-2.3;18.7)	-3.4 (-5.4;-1.7)^d^	2010-2012	-7.9 (-10.4;-5.0)^d^	-5.8 (-6.3;-5.4)^d^
	2013-2021	-5.9 (-11.4;-4.7)^d^		2012-2015	0.6 (-1.8;2.2)	
	–	–	–	2015-2021	-8.2 (-9.5;-7.5)^d^	
Santa Catarina	2010-2019	-6.5 (-7.7;-3.4)^d^	-9.7 (-12.0;-7.7)^d^	2010-2015	-2.6 (-3.6;-0.4)^d^	-9.4 (-10.3;-8.7)^d^
	2019-2021	-23.0 (-33.7;-10.0)^d^		2015-2019	-7.4 (-9.5;-5.3)^d^	
	–	–	–	2019-2021	-27.5 (-31.9;-20.9)^d^	
Rio Grande do Sul	2010-2016	-3.4 (-4.9;-0.3)^d^	-6.5 (-7.5;-5.6)^d^	2010-2013	4.7 (1.3;12.0)^d^	-4.3 (-5.3;-3.3)^d^
	2016-2021	-10.0 (-15.2;-7.8)^d^		2013-2019	-4.3 (-5.6;-2.6)^d^	
	–	–	–	2019-2021	-16.3 (-21.2;-10.0)^d^	
**Midwest**	2010-2019	1.4 (0.1;9.2)^d^	-1.4 (-3.5;1.4)	2010-2017	0.7 (-1.2;3.7)	-3.6 (-5.2;-2.2)^d^
	2019-2021	-13.0 (-23.7;-1.0)^d^		2017-2021	-10.5 (-18.3;-6.6)^d^	
Mato Grosso do Sul	2010-2021	4.4 (-1.6;11.7)	4.4 (-1.6;11.7)	2010-2021	-1.6 (-5.6;2.7)	-1.6 (-5.6;2.7)
Mato Grosso	2010-2013	-11.4 (-23.5;-4.4)^d^	-4.8 (-6.7;-3.4)^d^	2010-2012	-11.6 (-21.6;4.0)	-5.1 (-7.0;-3.0)^d^
	2013-2017	13.1 (7.2;22.1)^d^		2012-2016	10.7 (1.9;22.0)^d^	
	2017-2021	-15.5 (-23.1;-10.5)^d^		2016-2021	-13.7 (-20.4;-9.9)^d^	
Goiás	2010-2015	4.9 (-0.4;22.7)	-2.4 (-6.9;0.7)	2010-2017	0.4 (-3.2;33.3)	-5.0 (-9.7;0.5)
	2015-2021	-8.2 (-27.8;-4.4)^d^		2017-2021	-13.7 (-40.2;-5.8)^d^	
Distrito Federal	2010-2021	-0.6 (-3.8;2.6)	-0.6 (-3.8;2.6)	2010-2012	25.9 (6.8;55.4)^d^	-1.8 (-5.2;2.0)
	–	–	–	2012-2019	-2.5 (-6.1;1.0)	
	–	–	–	2019-2021	-21.5 (-34.7;-7.8)^d^	
**B** **razil**	2010-2019	-0.3 (-1.0;0.7)	-3.6 (-4.7;-2.9)^d^	2010-2019	-1.7 (-2.3;-1.0)^d^	-5.1 (-5.8;-4.4)^d^
	2019-2021	-17.5 (-23.2;-10.7)^d^		2019-2021	-18.8 (-22.7;-12.3)^d^	

a) APC: Annual percentage change; b) 95%CI: 95% confidence interval (lower limit; upper limit); c) AAPC: Average annual percentage change; d) Statistically significant value.

In the population aged between 35 and 59 years, there was a falling trend in the dual infection incidence coefficients in Brazil as a whole and in all its macro-regions. None of the states presented positive AAPC, with Santa Catarina (AAPC = -9.4: 95%CI -10.3;-8.7) being the state that showed the greatest falling trend in TB-HIV incidence in this age group; ten FU recorded periods with positive APCs, such as Pará, Maranhão and Sergipe (Table 4).

## DISCUSSION

This TB-HIV coinfection time series study revealed that the states of Rio Grande do Sul, Amazonas, Pernambuco and Santa Catarina had the highest levels of incidence in Brazil between 2010 and 2021. Falling trends were seen mainly in the FU in the Southern and Southeastern regions. Increases in incidence were recorded, especially in the male population and in those aged 18 to 34 years. There was a downward trend in most FU during the COVID-19 pandemic.

The Brazilian response to the HIV and TB epidemics, historically, is related to political and budgetary issues. It is known that since 2013 Brazil has faced unprecedented socioeconomic adversities, the rise of inequalities and the impact of these obstacles on the Brazilian National Health System (*Sistema Único de Saúde* - SUS),^12^ associated with aspects of regional disparity in the distribution of and access to services.^11,^This is a reality to be considered when interpreting the different trends found in this study.

It is essential to recognize the development of collaborative strategies between TB and HIV programs in Brazil, such as:^5,^ ^16^ ^,17^ recommending antiretroviral therapy (ART) for people with HIV, regardless of their lymphocyte count, with effect from 2011; incorporation of rapid molecular testing for TB into the health care network in 2014; and strengthening the detection and treatment of TB infection, especially in people living with HIV, following the publication of the surveillance protocol in 2018.

Internationally, in the United Kingdom, a falling trend in TB-HIV coinfection incidence was also seen between 2000 and 2014.^18^ That fall was linked to the increase in the lymphocyte count threshold for starting ART in 2008, contributing to the improvement of quality of life of people with HIV and reducing susceptibility to TB.^18^ In Brazil, it is assumed that the indication of ART for all people with HIV may have influenced the reduction in cases of coinfection with TB.

The decrease in the levels of TB-HIV coinfection incidence may also be related to the expansion of TB infection diagnosis and treatment actions, both among the general population and among people living with HIV. This is one of the fundamental strategies for eliminating TB as a public health problem, in Brazil and around the world,^19^ as it can result in a reduction in the incidence of TB disease cases and contribute to interrupting the Koch bacillus transmission chain.^20^

It is also important to highlight the crucial role that pre-exposure prophylaxis (PrEP) can play in addressing the HIV epidemic. It is a fact that the expansion of prevention options in Brazil, since 2017, such as PrEP and methods from the perspective of combined prevention, can culminate in effective control of infection.^21^ Therefore, it is inferred that the reduction in new HIV cases could reduce the incidence of TB coinfection, due to the smaller number of potentially susceptible people.

The growing trend in TB-HIV coinfection, however, is linked to (i) the greater circulation of etiological agents, which favors the transmission of infections, and/or (ii) the greater supply of tests to detect HIV and TB, which increases the number of people diagnosed. In Brazil, testing for HIV in people with TB and investigating TB in people with HIV (dual testing) is a strategy of the health care network, especially in Primary Health Care and Specialized Care Services.^16^

In this sense, it is considered that the expansion of TB testing among people with HIV can lead to substantial increases in the number of cases of coinfection, culminating in rising trends in incidence. As an example, a study carried out in a region of Ghana showed that the strengthening of collaborative actions between TB and HIV control programs, such as the provision of tests, resulted in significant periods of increased coinfection in the time series from 2008 to 2018.^22^

The dual testing policy is particularly relevant in Brazil, given the high number of people who discover their HIV infection only as a result of TB. In 2020, data resulting from linkage between the SINAN, SIM, SISCEL and SICLOM databases showed that 47.9% of registered coinfection cases were diagnosed with HIV due to TB.^19^ This is a warning sign for possible late diagnosis of HIV, which affects just over a quarter of the cases of infection registered in Brazil.^23^

It should be highlighted that identification of a stationary trend in TB-HIV coinfection raises another alert, regarding possible weaknesses in TB care, such as ineffective contact assessment and active tracing. This situation results in an epidemiological plateau, given that, even with the diagnosis and treatment of people with TB disease, those with TB infection can go unnoticed and, eventually, progress to the active form,^20^ sustaining the TB transmission chain among people living with HIV.

In addition to territorial disparities, there was a significant increase in cases of TB-HIV coinfection in the population aged 18 to 34. In the Brazilian context, young people have accounted for the majority of cases of HIV infection, mainly because of risky health practices, such as (i) starting one’s sex life early, (ii) inconsistent use of prevention methods, such as condoms and PrEP, (iii) low level of education, (iv) intercourse with multiple partners and (v) use of alcohol and drugs, among others.^24^

Furthermore, issues of sex, gender identity and sexual orientation must be considered, which may be related to the risk of HIV infection in Brazil, causing gay men and other men who have sex with men (MSM) to be considered as key populations for addressing the epidemic.^25^ Added to this, there is the issue of male vulnerability to TB: they are the most affected by TB infection in Brazil, accounting for 70% of new cases registered between 2020 and 2022.^26^

This intersection highlights the need for HIV and TB programs to promote strategies that take into account the social issues linked to infection. For example, the Interministerial Committee for the Elimination of Tuberculosis and Other Socially Determined Diseases (*Comitê Interministerial para a Eliminação da Tuberculose e de Outras Doenças Determinadas Socialmente* - CIEDDS) has been set up in Brazil, by Decree No. 11494, dated April 17, 2023. The CIEDDS aims to promote intersectorial actions to eliminate TB and other socially determined diseases, such as HIV.

In addition to the strategies adopted to control TB-HIV coinfection in Brazil, the aim is to develop specific actions, from the perspective of holistic care: organization of the line of care, for timely initiation and adherence to ART; dual testing and reduction of late diagnosis of infections; access, linkage and retention of affected individuals, for follow-up in health services; and detection and treatment of TB among the general population and among those living with HIV – including preventive treatment.

In Latin America and the Caribbean, diagnosis and treatment policies, such as those described above, have been adopted in more than 80% of countries; however, there are flaws that weaken the monitoring of TB-HIV coinfection, such as the lack of simultaneous integration of case notification, which affects the quality of care provided.^27^ Nevertheless, recognition must be given to the effort made by the Ministry of Health in carrying out periodic health information system linkage to improve information at the national level.

The COVID-19 pandemic may have accentuated the obstacles faced by the SUS, as it hampered access to diagnosis and compromised surveillance actions, resulting in a drop in TB-HIV coinfection incidence rates, as identified in this study. The pandemic also made TB monitoring and treatment difficult and interrupted follow-up activities for people living with HIV in Brazil,^28,^ ^29^ which would explain the falling trends seen at the end of the time series (2019 to 2021).

It should also be noted that interruptions in the provision of TB and HIV services, resulting from the emergence of the COVID-19 pandemic, could result in significant increases in morbidity and mortality associated with infection in the coming years.^30^ As such, in addition to underdetection and/or underreporting of cases of dual infection in the Brazilian scenario, another warning is highlighted in this report, given the possibility of there being recorded a substantial number of deaths and years of potential life lost as a result of TB and/or HIV.^30^

It is important to point out that this research has limitations. Firstly, the use of secondary data may be subject to incorrect and/or incomplete filling out, in addition to different levels of underreporting in the territories, so the findings presented here may be underestimated in certain locations. Other limitations of this study would be the fact that (i) the models were not adjusted for confounding factors, which could influence the trends, (ii) the non-inclusion of cases from the entire Brazilian population, being restricted only to people aged 18 to 59, and (iii) the data being aggregated at the state level, preventing the understanding of dynamics at the municipal level. 

Moreover, it should be noted that, although linkage between the SINAN, SIM, SISCEL and SICLOM databases results in an annual increase of around a thousand cases of TB-HIV coinfection, in relation to data produced by the SINAN alone,^5^ the greater or lesser involvement of local surveillance services in notifying cases, both TB and HIV, may influence the downward or upward trends found in this study. All of this confirms the importance of adequate recording and timely notification of cases of TB-HIV coinfection to ensure quality information.

In short, demographic and territorial disparities were evident in the trends of TB-HIV coinfection incidence in Brazil. Rising trends were seen, especially in the North and Northeast regions, among males and in the population aged 18 to 34 years. There was a reduction in incidence rates for most FU between 2019 and 2021, which points to the possible effects of the COVID-19 pandemic on diagnosis of TB and HIV.

Without disregarding the possible impact of the pandemic on the progress achieved so far, at a national level, the findings of this study can contribute to the planning of actions to control TB-HIV coinfection in the most affected territories and groups in Brazil. Therefore, the information presented can support the implementation or readjustment of state and national public policies, with a view to reversing the epidemiological scenario and achieving better conditions in Brazilian public health.

## References

[1] Rewari BB, Kumar A, Mandal PP, Puri AK (2021). HIV TB coinfection - perspectives from India. Expert Rev. Respir. Med.

[2] Cruz DKA, Nóbrega AA, Montenegro MMS, Pereira VOM (2022). The Sustainable Development Goals and data sources for monitoring
goals in Brazil. Epidemiol Serv Saude.

[3] World Health Organization (2022). Global tuberculosis report, 2022 [Internet].

[4] Torpey K, Agyei-Nkansah A, Ogyiri L, Forson A, Lartey M, Ampofo W (2020). Management of TB/HIV co-infection: the state of the
evidence. Ghana Med J.

[5] Ministério da Saúde (BR) (2021). Boletim epidemiológico: panorama epidemiológico da coinfecção TB-HIV no
Brasil, 2020 [Internet].

[6] Cavalin RF, Pellini ACG, Lemos RRG, Sato APS (2020). Rev Saude Publica.

[7] Rossetto M, Maffacciolli R, Rocha CMF, Oliveira DLLC, Serrant L (2019). Tuberculosis/HIV/AIDS coinfection in Porto Alegre, RS/Brazil -
invisibility and silencing of the most affected groups. Rev Gaucha Enferm.

[8] Santos LFS, Carneiro PHV, Serra MAAO, Santos LH, Andrade HLP, Pascoal LM (2022). Tuberculosis/HIV co-infection in Northeastern Brazil: prevalence
trends, spatial distribution, and associated factors. J Infect Dev Ctries.

[9] Reis AA, Alecrim TFA, Zerbetto SR, Palha PF, Ruggiero CM, Protti-Zanatta ST (2022). Live/cope with tuberculosis/HIV and the meanings represented by
the illness process: a discourse analysis. Cienc Cuid Saude.

[10] Albuquerque MV, Ribeiro LHL (2021). Inequality, geographic situation, and meanings of action in the
COVID-19 pandemic in Brazil. Cad Saude Publica.

[11] Albuquerque AC, Cesse EAP, Felisberto E, Samico IC, Frias PG (2019). Avaliação de desempenho da regionalização da vigilância em saúde em seis
regiões de saúde brasileiras.

[12] Pinto LF, Quesada LA, D’Avila OP, Hauser L, Gonçalves MR, Harzheim E (2021). Primary Care Assessment Tool: regional differences based on the
National Health Survey from Instituto Brasileiro de Geografia e
Estatística. Cien Saude Colet.

[13] Ministério da Saúde (BR) (2023). Estudo de estimativas populacionais por município, idade e sexo
2000-2021 – Brasil [Internet].

[14] National Cancer Institute (USA) (2023). Surveillance Research Program.

[15] Agostini R, Rocha F, Melo E, Maksud I (2019). The Brazilian response to the HIV/AIDS epidemic amidst the
crisis. Cien Saude Colet.

[16] Ministério da Saúde (BR) (2017). Brasil livre da tuberculose: plano nacional pelo fim da tuberculose como
problema de saúde pública [Internet].

[17] Ministério da Saúde (BR) (2018). Protocolo de vigilância da infecção latente pelo *Mycobacterium
tuberculosis* no Brasil.

[18] Winter JR, Stagg HR, Smith CJ, Lalor MK, Davidson JÁ, Brown AE (2018). Trends in, and factors associated with, HIV infection amongst
tuberculosis patients in the era of anti-retroviral therapy: a retrospective
study in England, Wales and Northern Ireland. BMC Med.

[19] Ministério da Saúde (BR) (2023). Boletim epidemiológico da coinfecção TB-HIV, 2022 [Internet].

[20] Cohen A, Mathiasen VD, Schön T, Wejse C (2019). The global prevalence of latent tuberculosis: a systematic review
and meta-analysis. Eur Respir J.

[21] Zucchi EM, Grangeiro A, Ferraz D, Pinheiro TF, Alencar T, Ferguson L (2018). Da evidência à ação: desafios do Sistema Único de Saúde para
ofertar a profilaxia pré-exposição sexual (PrEP) ao HIV às pessoas em maior
vulnerabilidade. Cad Saude Publica.

[22] Salisu HM, Ojule IN, Adeniji FO, Kwakye GK (2022). Prevalence and trend of TB/HIV co-infection in Suhum
municipality, Ghana. PLoS Glob Public Health.

[23] Ministério da Saúde (BR) (2023). Indicadores e dados básicos de monitoramento clínico de HIV.

[24] Bossonario PA, Ferreira MRL, Andrade RLP, Sousa KDL, Bonfim RO, Saita NM (2022). Risk factors for HIV infection among adolescents and the youth: a
systematic review. Rev Lat Am Enfermagem. Rev Lat Am Enfermagem.

[25] Ministério da Saúde (BR) (2018). Agenda estratégia para ampliação do acesso e cuidado integral das
populações-chave em HIV, hepatites virais e outras infecções sexualmente
transmissíveis [Internet].

[26] Ministério da Saúde (BR) (2023). Boletim epidemiológico da tuberculose, 2023 [Internet].

[27] Moreno R, Ravasi G, Avedillo P, Lopez R (2020). Tuberculosis and HIV coinfection and related collaborative
activities in Latin America and the Caribbean. Rev Panam Salud Publica.

[28] Berra TZ, Ramos ACV, Alves YM, Tavares RBV, Tartaro AF, Nascimento MC (2022). Impact of COVID-19 on tuberculosis indicators in Brazil: a time
series and spatial analysis study. Trop Med Infect Dis.

[29] Matsuda EM, Oliveira IP, Bao LB, Manzoni FM, Campos NC, Varejão BB (2022). Impact of COVID-19 on people living with HIV-1: care and
prevention indicators at a local and nationwide level, Santo André,
Brazil. Rev Saude Publica.

[30] Hogan AB, Jewell BL, Sherrard-Smith E, Vesga JF, Watson OJ, Whittaker C (2020). Potential impact of the COVID-19 pandemic on HIV, tuberculosis,
and malaria in low-income and middle-income countries: a modelling
study. Lancet Glob.

